# M2‐polarized tumor‐associated macrophage‐secreted exosomal lncRNA NEAT1 upregulates galectin‐3 by recruiting KLF5 and promotes HCC immune escape

**DOI:** 10.1002/ccs3.12060

**Published:** 2024-12-23

**Authors:** Wei Yuan, Qigang Sun, Xiaodan Zhu, Bo Li, Yongping Zou, Zhehao Liu

**Affiliations:** ^1^ Department of Emergency Surgery Hainan General Hospital Hainan Affiliated Hospital of Hainan Medical University Haikou China; ^2^ Department of Hepatobiliary and Pancreatic Surgery Hainan General Hospital Hainan Affiliated Hainan Hospital of Hainan Medical College Haikou China

**Keywords:** exosomes, galectin-3, hepatocellular carcinoma, immune escape, KLF5, NEAT1, tumor‐associated macrophages

## Abstract

HCC cell immune escape is a critical element in the evolution of HCC malignancy. Herein, the regulatory mechanism of lncRNA NEAT1 in regulating HCC immune escape was investigated. Exosomes were isolated from M2 TAMs using ExoQuick‐TC. Then, HCC cells were incubated with M2 TAMs‐derived exosomes (M2‐exos). The activation of perforin^+^CD8^+^ T cells was measured using flow cytometry. The secretion of IFN‐γ was assessed using ELISA. Cell viability and migration were detected using CCK8 and Transwell assays, respectively. RIP and RNA pull‐down assays were used to investigate the link between NEAT1 and KLF5. ChIP and dual‐luciferase reporter assays were used to investigate the interaction between KLF5 and the LGALS3 promoter. Our results showed that NEAT1, KLF5 and galectin‐3 were overexpressed in HCC tissues. M2‐exos treatment promoted HCC proliferation, migration, and immune escape. It was found that NEAT1 was enriched in M2‐TAMs and M2‐exos. M2‐exos facilitated HCC immune escape, whereas NEAT1 silencing reversed this effect. NEAT1 upregulated galectin‐3 in HCC cells by recruiting KLF5. Mechanically, M2‐TAM‐derived exosomal NEAT1 induced HCC immune escape by upregulating KLF5/galectin‐3 axis. M2‐TAM‐derived exosomal NEAT1 upregulated galectin‐3 in HCC cells by recruiting KLF5 to promote perforin^+^CD8^+^ T cell depletion and further accelerate HCC immune escape.

## Graphical abstract

Exosomal lncRNA NEAT1 secreted by M2 polarized tumor‐associated macrophages promotes HCC immune escape by recruiting KLF5 and upregulating galectin‐3.

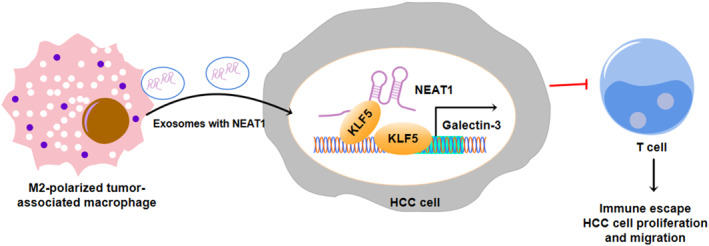

## 1 INTRODUCTION

Hepatocellular carcinoma (HCC) is the fifth most prevalent malignancy and the second major cause of cancer death worldwide.^1^ Currently, the primary therapeutic options for HCC are ineffective in controlling tumor recurrence and metastasis.^2^ Therefore, the ideal therapy for HCC should not only remove the tumor in situ but also remove residual tumor cells by activating the immune system to prevent tumor metastasis and recurrence.^3^ Tumor cells may resist immune system detection and assault via several pathways, which constitutes a key strategy for tumor survival and progression, and this process is known as tumor immune escape.^4^ Therefore, inhibiting immune escape is crucial to improve the efficacy of immunotherapy for HCC. Tumor‐associated macrophages (TAMs) are major infiltrating noncancerous cells and are closely associated with tumor proliferation, metastasis, invasion, and immune escape in the tumor microenvironment (TME).^5^ Due to the heterogeneity of macrophages, TAMs can be divided into M1‐TAMs and M2‐TAMs.

Most macrophages exhibit the M1 phenotype early and inhibit tumor growth. In contrast, as tumor progression continues, macrophages gradually converge to the M2 phenotype, contributing to tumor immune escape.^6^, ^7^ As reported, M2‐TAMs participate in tumor progression by facilitating immune escape through facilitating CD8^+^ T cell exclusion.^8^ It's suggested that exploring the mechanisms involved is of great significance in addressing the issue of tumor immune escape in HCC.

Noncoding RNAs longer than 200 nucleotides (nts) are referred to as long noncoding RNAs (lncRNAs),^9^ which are involved in tumorigenesis,^10^ angiogenesis,^11^ and tumor metastasis.^12^ LncRNAs have a major role in the development of tumors and immune escape. As evidence, lncRNA FENDRR upregulation inhibited HCC cell proliferation and the Treg‐mediated immune escape.^4^ LncRNA nuclear paraspeckle assembly transcript 1 (NEAT1) aided in the growth of several malignant cancers, including HCC.^13^, ^14^, ^15^ Kou et al. illustrated that NEAT1 was upregulated in HCC tissues, and its silencing restrained HCC cell proliferation and promoted cell apoptosis.^13^ It was described that M2‐TAM‐secreted exosomal NEAT1 could promote ovarian cancer immune escape.^6^ However, the role of M2‐TAM‐secreted exosomal NEAT1 in regulating HCC immune escape remains unclear and deserves further research.

Krüppel‐like factor 5 (KLF5), as a member of the KLF transcription factor family, is closely related to cancer progression by regulating the expression of downstream targets.^16^ KLF5 is widely reported to be overexpressed in HCC, and its downregulation repressed HCC cell malignant behaviors.^17^, ^18^ A previous study displayed that KLF5 knockdown elevated the number and functionality of intratumoral antitumor T cells,^19^ suggesting that KLF5 may have immunosuppressive effects to contribute to tumor cell immune escape. Notably, Ma et al. illustrated that KLF5 contributed to tumorigenesis in gastric cancer by transcriptionally activating NEAT1.^20^ Herein, KLF5 was predicted to have potential binding sites to NEAT1 using the RPISeq database. All these pieces of evidence suggest that NEAT1 may promote HCC immune escape by recruiting KLF5, which has never been reported before.

Galectin‐3, encoded by the LGALS3 gene, is a multifunctional protein that has been linked to cancer development and metastasis.^21^ It has been widely described that galectin‐3 is highly expressed in multiple types of cancer, including HCC.^22^ Recent research has shed light on the role of galectin‐3 as a ligand for lymphocyte activation gene‐3 (LAG3), which jointly affects T cell function with LAG3, thereby promoting immune escape of tumor cells.^23^ As evidence has shown that galectin‐3 suppressed anti‐tumor immune responses by decreasing plasmacytoid dendritic cell growth and lowering CD8^+^ T cell proliferation via LAG3.^24^ Herein, KLF5 was predicted to potentially bind to the LGALS3 promoter by using the JASPAR database. It suggests that KLF5 may promote T cell inhibition in HCC through transcriptional activation of galectin‐3.

It's hypothesized that M2‐TAM‐secreted exosomal NEAT1 upregulated galectin‐3 by recruiting KLF5, thus promoting immune escape in HCC cells. These findings lay the theoretical groundwork for the development of innovative HCC treatments.

## 2 MATERIALS AND METHODS

### 2.1 Clinical sample collection

Post‐operatively, 10 HCC tumor specimens and adjacent normal tissues were taken from HCC patients at Hainan General Hospital, Hainan Affiliated Hospital of Hainan Medical University. Pathology clearly diagnosed the patients, and they were not treated. The Ethics Committee at Hainan General Hospital, Hainan Affiliated Hospital of Hainan Medical University approved this study, and all subjects signed informed permission.

### 2.2 Cell culture and treatment

ATCC (VA, USA) provided the human monocytes (THP‐1 cells) and HCC cells (HepG2 and MHCC97L cells). All cells were grown in DMEM (Gibco, MD, USA) mixed with 10% FBS (Gibco) with 5% CO_2_ at 37°C. For exosome treatment, HCC cells were incubated with 100 μg/ml exosomes for 12 h. Millicell cell culture inserts with a pore size of 1.0 μm (Millipore, MA, USA) were sown at the bottom of 6‐well plates containing M2‐TAMs, and 2 × 10^5^ HCC cells were seeded onto the inserts and cultured for 24 h. Blood was taken from 25 healthy volunteers and used to isolate peripheral blood mononuclear cells (PMBCs) using the Ficoll‐Hypaque density gradient method (Sigma‐Aldrich, MO, USA). The EasySep™ Human CD8^+^ T cell enrichment Kit (NovoBiotechnology, Beijing, China) was used to isolate CD8^+^ T cells from PBMCs. All donors provided informed consent. 10 U/mL IL‐2 was used to induce PMBCs, and then co‐cultured with HCC cells for subsequent experiments.

### 2.3 The induction and identification of M2 TAMs

To obtain M0 macrophages, THP‐1 cells were incubated with 100 ng/mL PMA (Sigma‐Aldrich) for 24 h. Macrophages were polarized in M2 macrophages by incubation with 20 ng/mL interleukin (IL)‐4 and IL‐13 (R&D Systems, IL, USA). The markers (CD206, CD163 and CCL22) were verified using flow cytometry and western blot.

### 2.4 Cell transfection

GenePharma (Shanghai China) also provide the short hairpin RNAs (sh‐NEAT1 and sh‐KLF5), the overexpression plasmid of galectin‐3 (oe‐Gal‐3) and their negative controls. When the cell density reached 50%–70%, cells were transfected with the vectors using Lipofectamine 3000 (Invitrogen, CA, USA). After transfection for 48 h, RNA was collected for qRT‐PCR to verify the transfection efficiency.

### 2.5 Cell counting kit‐8 (CCK‐8) assay

Cells were cultured in 96‐well plates (5 × 10^3^ cells/well) for 24 h and treated for 3 h with 10 μL CCK‐8 solution (Sangon, Shanghai, China). The absorbance at 450 nm was recorded on a microplate reader (BioTex, TX, USA). All experiments were biologically repeated at least three times.

### 2.6 Flow cytometry

Cell concentration was diluted to 1 × 10^7^/mL using PBS containing 10% FBS. Cells were then stained for 30 min with anti‐CD8 (Abcam, Cambridge, UK, 1:500, ab217344) and anti‐LAG3 (Abcam, 1:500, ab16074) in the dark. Following two washes, cells were suspended in 2 mL PBS containing 10% FBS and analyzed using flow cytometry (BD, NJ, USA).

### 2.7 Isolation and identification of exosomes

Exosomes were extracted using ExoQuick‐TC (System Bioscience, CA, USA). The markers (CD81, CD63 and TSG101) were verified using flow cytometry. To determine the size, the exosomes were submitted to NTA (Malvern Panalytical, Malvern, UK). Exosomes were loaded and treated for 1 min with phosphotungstic acid (Sigma‐Aldrich) before being examined using transmission electron microscopy (TEM) (HITACHI, Tokyo, Japan).

### 2.8 Exosome labeling and uptake

Exosomes resuspended in Diluent C were treated for 4 min with 4 μL PKH67 dye (Sigma‐Aldrich). HCC cells were incubated with labeled exosomes for 12 h. After that, the HCC cells were incubated with iFluor 555 WGA (Solarbio, Beijing, China) working solution at 37°C for 20 min. Then cells were fixed, labeled with DAPI and imaged using laser scanning confocal microscopy (Olympus, Tokyo, China).

### 2.9 Transwell assay

Cells were harvested and resuspended in serum‐free medium and then seeded onto the upper chamber (BD, NJ, USA) at a density of 1 × 10^4^ cells, whereas the bottom chamber was filled with DMEM containing 10% FBS (1000 μL). After 12 h, cells on the top chamber were removed, whereas cells on the surface of the lower chamber were fixed with 0.5% crystal violet. A microscope (Olympus) was used to image cells.

### 2.10 Enzyme linked immunosorbent assay (ELISA)

Interferon (IFN)γ level in PBMCs was detected by the ELISA kits purchased from Abcam (ab174443). All operations were carried out in strict accordance with the manuals. The OD values were recorded at 450 nm and analyzed by Origin 9.5 software.

### 2.11 Quantitative real‐time polymerase chain reaction (qRT‐PCR)

Total RNA was extracted using Trizol reagent (Invitrogen), and the NanoDrop 2000 was applied for RNA concentration and quality quantification. Total RNA (1 μg) was reversely transcribed into cDNA with PrimeScript™ RT Kit (Takara, Tokyo, Japan) and subjected to qRT‐PCR assay using SYBR Green Master Mix (Takara). As the reference gene, GAPDH was employed. The relative quantification for target genes was calculated using the 2^−∆∆CT^ method. The primers used in the study were listed as follows (5′‐3′):

NEAT1 (F): GGCACAAGTTTCACAGGCCTACATGGG.

NEAT1 (R): GCCAGAGCTGTCCGCCCAGCGAAG.

GAPDH (F): AGGTCGGTGTGAACGGATTTG.

GAPDH (R): GGGGTCGTTGATGGCAACA.

### 2.12 Western blot

A BCA kit from Beyotime (Shanghai, China) was used to measure the proteins after they had been separated using RIPA (Beyotime). Subsequently, total protein (20 μg) was isolated by 10% SDS‐PAGE and transferred to a PVDF membrane (Millipore). The membranes were blocked and incubated with antibodies against KLF5 (Abcam, 1:1000, ab137676), galectin‐3 (Abcam, 1:5000, ab76245), CD206 (Abcam, 1:2000, ab64693), CD163 (Abcam, 1:1000, ab182422), CCL22 (Abcam, 1:1000, ab9847), CD81 (Abcam, 1:1000, ab79559), CD63 (Abcam, 1:1000, ab134045), TSG101 (Abcam, 1:1000, ab125011) and GAPDH (Abcam, 1:5000, ab8245), overnight, then hybridized for 60 min with the secondary antibody (ab7090). The bands were examined by ECL (Beyotime). The grayscale of those protein bands was analyzed by using Image *J*.

### 2.13 RNA binding protein immunoprecipitation (RIP) assay

The total RNA was incubated with KLF5 antibody (Abcam, 1:30, ab137676) or IgG antibody (Abcam, 1:100, ab109489) and treated for 1 h with protein *A*/*G* magnetic beads (Thermo Fisher Scientific). RNA was purified from the RNA‐bead‐antibody complex and analyzed using qRT‐PCR.

### 2.14 RNA pull‐down assay

NEAT1‐sense or NEAT1‐antisense RNA was transcribed, biotin‐labeled (Roche, Mannheim, Germany) and purified. In the gentle lysis buffer containing 80 U/mL RNasin (Promega), about 2 × 10^7^ cells were lysed. Cell extract was incubated with biotinylated RNA for 1 h, followed by incubation with washed streptavidin‐coupled agarose beads (Invitrogen) for 1 h. Beads were washed, and the retrieved protein was assessed using western blot.

### 2.15 Dual‐luciferase reporter assay

LGALS3 promoter fragments containing KLF5 binding site (LGALS3‐WT) or the mutated binding site (LGALS3‐MUT) were amplified by PCR and inserted into the pGL3 reporter plasmids (Promega, WI, USA). Then, cells were co‐transfected with LGALS3‐WT or LGALS3‐MUT plasmids and oe‐NC or oe‐KLF5 using Lipofectamine™ 3000, and the luciferase activity was subsequently tested.

### 2.16 Chromatin immunoprecipitation (ChIP) assay

Cells were fixed with 1% formaldehyde for 5 min to induce DNA–protein cross‐linking. The cell lysate was then ultrasonically treated to produce chromatin fragments, and incubated with anti‐KLF5 (Abcam, 1:60, ab277773) or anti‐IgG (Abcam, 1:100, ab172730) at 4°C overnight. DNA that binds to KLF5 was immunoprecipitated using Pierce protein *A*/*G* beads (Thermo Fisher Scientific), and the cross‐linking was eliminated. The precipitated DNA was then analyzed using agarose gel electrophoresis.

### 2.17 Animal experiments

4 week‐old male C57BL/6 mice were purchased from Beijing Vital River Laboratory Animal Technology Co., Ltd (Beijing, China), and randomized into the control group, M2‐exo group and M2‐exo‐shNEAT1 group, with a total of five mice in each group. For xenograft studies, HepG2 and HCCLM3 cells (5 × 10^6^ cells per mouse) were injected subcutaneously into the right flanks of mice. Every 5 days, measurements of the size of the tumor were recorded, and the volume of the tumor was determined by applying the following formula: length×width^2^ × 0.52. Starting on day 7, the tumor‐implanted mice received intraperitoneal injections of either M2‐exo (40 mg/kg), M2‐exo‐shNEAT1 (40 mg/kg), or saline every three days, and they were euthanized 30 days after the initial injection. The Hainan Provincial People's Hospital Medical Ethics Committee authorized and oversaw the study.

### 2.18 Immunohistochemistry (IHC)

Following deparaffinization and antigen retrieval (Dako, CA, USA), the tumor tissue slices were then blocked and incubated overnight with antibodies against KLF5 (Abcam, 1:200, ab137676) and galectin‐3 (Abcam, 1:250, ab76245). The sections were then treated for 1 h with the secondary antibody (Abcam, 1:500, ab150077). The sections reacted with DAB solution, stained with hematoxylin, dried, and sealed with neutral gum. The photos were captured with an Olympus microscope (Tokyo, Japan).

### 2.19 Statistical analysis

All experimental data were analyzed using GraphPad Prism8 statistical software and presented as the mean ± SD. Data between two groups were compared by student's *t*‐test, and data among multiple groups were analyzed by one‐way ANOVA with Tukey's multiple comparisons test. *p* < 0.05 was considered as a statistically significant difference. All experiments were repeated at least 3 times.

## 3 RESULTS

### 3.1 NEAT1, KLF5 and galectin‐3 were highly expressed in HCC clinical samples

**FIGURE 1:**
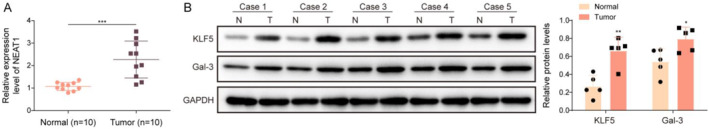
NEAT1, KLF5 and galectin‐3 were highly expressed in HCC clinical samples. HCC tumor tissues and adjacent normal tissues were collected from diagnosed HCC patients. (A) NEAT1 expression in tissues was detected by qRT‐PCR (*n* = 10). (B) Western blot was employed to determine KLF5 and galectin‐3 protein levels in tissues (*n* = 5). The measurement data were presented as mean ± SD. All data was obtained from at least three replicate experiments. *p < 0.05, **p < 0.01, ***p < 0.001.

10 HCC tumor specimens and adjacent normal tissues were collected from HCC patients post‐operatively. NEAT1 was considerably overexpressed in HCC tissues relative to adjacent normal tissues, as seen in Figure 1A. Furthermore, KLF5 and galectin‐3 protein levels in HCC tissues were significantly higher than in adjacent normal tissues (Figures 1B and S1A). Collectively, NEAT1, KLF5, and galectin‐3 were overexpressed in HCC tissues.

### 3.2 M2‐TAMs promoted HCC progression

**FIGURE 2:**
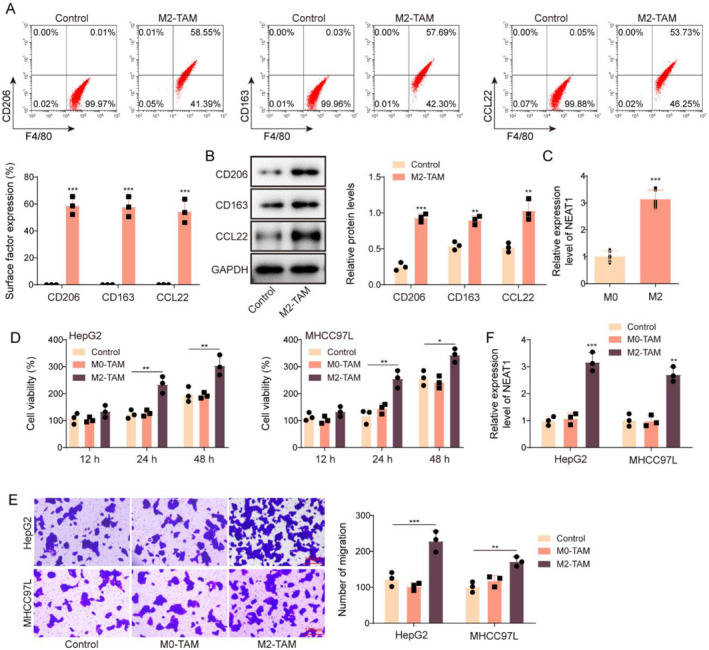
M2‐TAMs promoted HCC progression. THP‐1 cells were incubated with 100 ng/mL PMA for 24 h to obtain M0 macrophages, and M0 macrophages were incubated with 20 ng/mL IL‐4 and IL‐13 to obtain M2‐TAMs. (A‐B) The levels of M2 markers (CD206, CD163 and CCL22) were detected by flow cytometry and western blot. (C) NEAT1 expression in M0 and M2‐TAMs was assessed by qRT‐PCR. We subsequently co‐treated HCC cells with M0‐TAMs and M2‐TAMs, and the HCC cells were cultured in ordinary culture medium as the control group. (D) CCK8 assay was performed to detect HCC cell viability. (E) Cell migration was examined by Transwell assays (Scale bars = 100 μm) (F) NEAT1 expression in HCC cells was detected by qRT‐PCR. The measurement data were presented as mean ± SD. All data was obtained from at least three replicate experiments. **p < 0.01, ***p < 0.001.

M2‐TAMs have been reported to have clear cancer‐promoting functions.^25^ Herein, THP‐1 cells were treated for 24 h with 100 ng/mL PMA to generate M0 macrophages, which were subsequently incubated with 20 ng/mL IL‐4 and IL‐13 to generate M2‐TAMs. As shown in Figures 2A–B, M2‐TAMs presented higher levels of M2 markers (CD206, CD163 and CCL22) than the M0 macrophages group. In addition, NEAT1 was markedly upregulated in M2‐TAMs compared with M0 macrophages (Figure 2C). We subsequently co‐treated HCC cells with M2‐TAMs, and HCC cells in the control group were cultured in an ordinary culture medium. In addition, M0‐TAMs were used as the negative control. Functional experiments showed that M2‐TAM treatment remarkably facilitated HCC cell viability and migration compared with the control and M0‐TAMs groups (Figure 2D–E). It also turned out that NEAT1 expression in HCC cells was significantly elevated by M2‐TAMs treatment (Figure 2F). Collectively, NEAT1 was overexpressed in M2‐TAMs, and M2‐TAMs treatment could promote HCC cell viability and migration.

### 3.3 Extraction and identification of M2‐TAM‐derived exosomes

**FIGURE 3:**
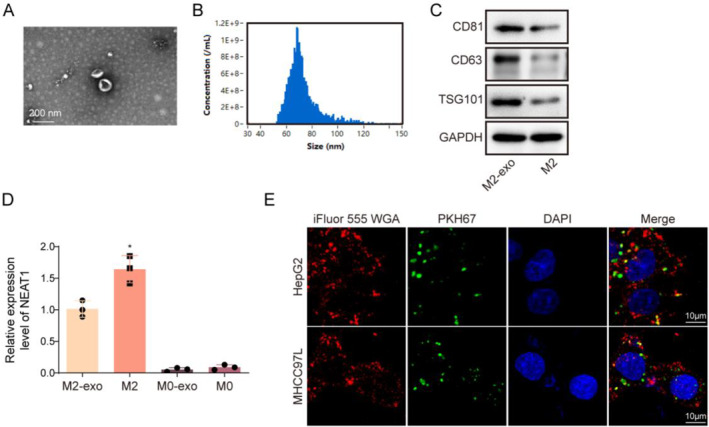
Extraction and identification of M2‐TAM‐derived exosomes. (A) The ultrastructure of exosomes was examined using TEM (Scale bar = 200 nm). (B) Exosomes were subjected to nanoparticle tracking analysis to detect size. (C) CD81, CD63 and TSG101 levels in M2‐TAMs, M2‐exos were elevated using western blot. (D) NEAT1 expression in M2‐TAMs, M2‐exos, M0‐TAMs, and M0‐exos was elevated using qRT‐PCR. (E) Immunofluorescence was employed to analyze the uptake of PKH67‐labelled exosomes by HCC cells, iFluor 555 WGA is used to label cell membranes (Scale bar = 10 μm). The measurement data were presented as mean ± SD. All data was obtained from at least three replicate experiments. *p < 0.05.

Exosomes were isolated from M2‐TAMs. Exosomes possessed distinctive morphological properties under TEM and a size of 55–150 nm, the polydispersity index is 0.36 ± 0.05, and the zeta potential is −22.73 ± 0.91 mV (Figure 3A–B). Results from western blot subsequently demonstrated that exosomes were CD81^+^, CD63^+^, and TSG101^+^ (Figure 3C). It was found that NEAT1 was both highly expressed in M2‐TAMs and M2‐TAM‐derived exosomes (M2‐exo). Moreover, compared with the M2‐exo group, the level of NEAT1 in M2‐TAM was higher. However, NEAT1 was observed lowly expressed in both M0‐TAMs and M0‐exo (Figure 3D). After incubation with HCC cells, PKH67‐labelled exosomes presented red fluorescence in HCC cells (Figure 3E), showing that exosomes could be taken up by HCC cells. Taken together, exosomes were successfully isolated from M2‐TAMs.

### 3.4 M2‐TAM‐derived exosomal NEAT1 promoted immune escape in HCC

**FIGURE 4:**
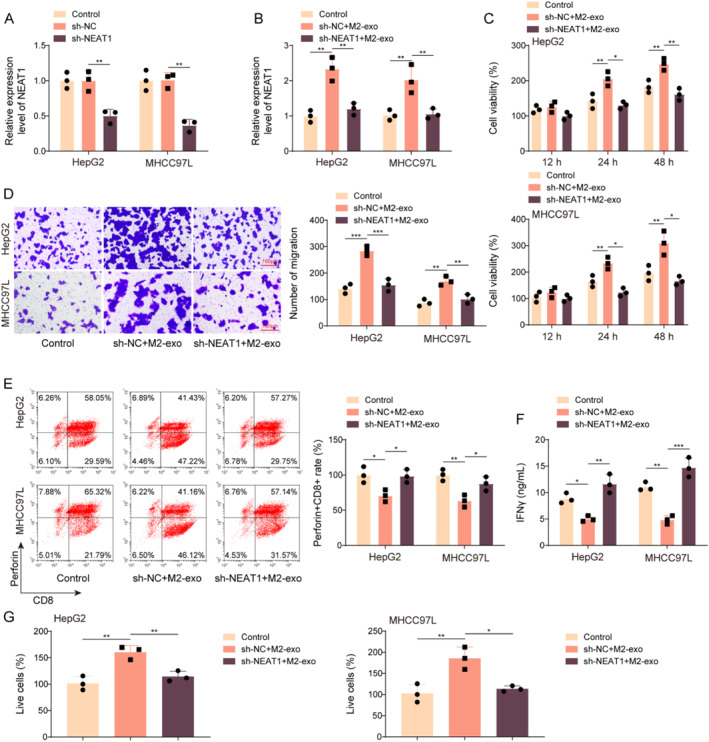
M2‐TAM‐derived exosomal NEAT1 prompted HCC immune escape. (A) M2‐TAMs were transfected with sh‐NC or sh‐NEAT1, and NEAT1 expression in M2‐TAMs and M2‐exos was assessed by qRT‐PCR. HCC cells were incubated with exosomes derived from M2‐TAMs transfected with sh‐NC or sh‐NEAT1. (B) NEAT1 expression in HCC cells was detected by qRT‐PCR. (C) CCK8 assay was employed to detect HCC cell viability. (D) Cell migration was measured by Transwell assays (Scale bars = 100 μm). CD8^+^ T cells isolated from PBMCs were co‐cultured with the above‐grouped HCC cells. (E) The activation of perforin^+^CD8^+^ T cells was measured by flow cytometry. (F) IFNγ level in CD8^+^ T cells was examined using ELISA. (G) CCK8 assay was employed to detect HCC cell viability. The measurement data were presented as mean ± SD. All data was obtained from at least three replicate experiments. *p < 0.05, **p < 0.01, ***p < 0.001.

To probe the role of NEAT1 in M2‐exo‐mediated the effect on HCC progression, we induced NEAT1 silencing in M2‐TAMs. NEAT1 expression in M2‐TAMs and M2‐exos was markedly reduced after sh‐NEAT1 transfection (Figure 4A). HCC cells were subsequently incubated with exosomes derived from M2‐TAMs transfected with sh‐NC or sh‐NEAT1. It turned out that M2‐exos treatment increased NEAT1 expression in HCC cells, while this change was eliminated by NEAT1 knockdown (Figure 4B). In addition, NEAT1 silencing reversed the promoting effect of M2‐exos on HCC cell viability and migration (Figure 4C–D). The EasySep™ Human CD8^+^ T cell enrichment Kit was used to isolate CD8^+^ T cells from PBMCs, and CD8^+^ T cells were co‐cultured with the above‐grouped HCC cells. Our results showed that M2‐exos treatment inhibited the activation of perforin^+^CD8^+^ T cells, whereas this effect was abolished by NEAT1 downregulation (Figure 4E). Additionally, IFNγ level in CD8^+^ T cells was decreased by M2‐exos treatment, which was abrogated by NEAT1 knockdown (Figure 4F). Moreover, M2‐exos treatment inhibited the inhibitory effect of CD8^+^ T cell co‐culture on HCC cell vitality while this effect was abolished by NEAT1 downregulation (Figure 4G). Taken together, M2‐exos prompted HCC immune escape by carrying NEAT1.

### 3.5 KLF5 directly bound to NEAT1 and galectin‐3

**FIGURE 5:**
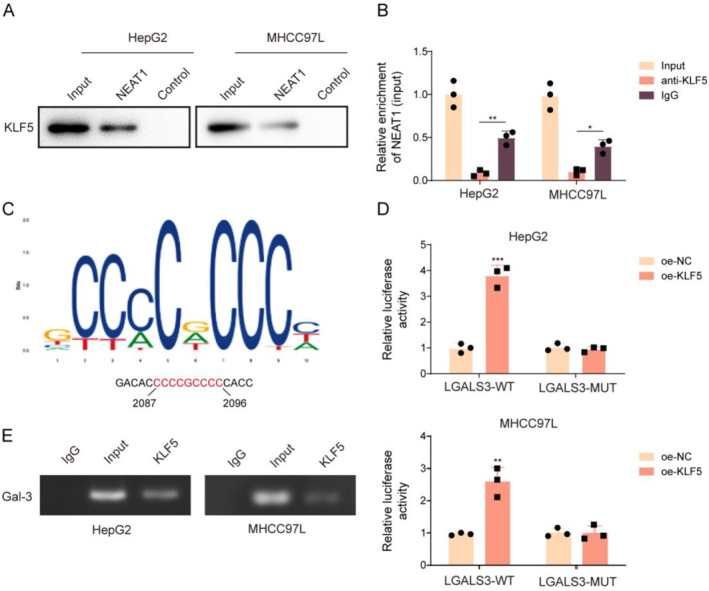
KLF5 is directly bound to NEAT1 and galectin‐3. (A‐B) The interaction between NEAT1 and KLF5 was analyzed by RNA pull‐down and RIP assays. (C) The potential binding sites between KLF5 and LGALS3 promoter were predicted using the JASPAR database. (D‐E) The interaction between KLF5 and LGALS3 promoter was analyzed by ChIP and dual‐luciferase reporter assays. The measurement data were presented as mean ± SD. All data was obtained from at least three replicate experiments. *p < 0.05, **p < 0.01, ***p < 0.001.

As revealed by RIP and RNA pull‐down assays, NEAT1 directly interacted with KLF5 (Figure 5A–B). In addition, using the JASPAR database, it was predicted that KLF5 had potential binding sites on the LGALS3 promoter (Figure 5C). ChIP results further showed that anti‐KLF5 antibody could significantly enrich the LGALS3 promoter in HCC cells (Figure 5D). Meanwhile, KLF5 overexpression boosted the luciferase activity of LGALS3‐WT but had no impact on LGALS3‐MUT (Figure 5E). All the above results suggested that KLF5 transcriptionally activated galectin‐3. In summary, both NEAT1 and galectin‐3 are directly bound with KLF5.

### 3.6 M2‐TAM‐derived exosomal NEAT1 upregulated galectin‐3 by recruiting KLF5 to promote immune escape in HCC

**FIGURE 6:**
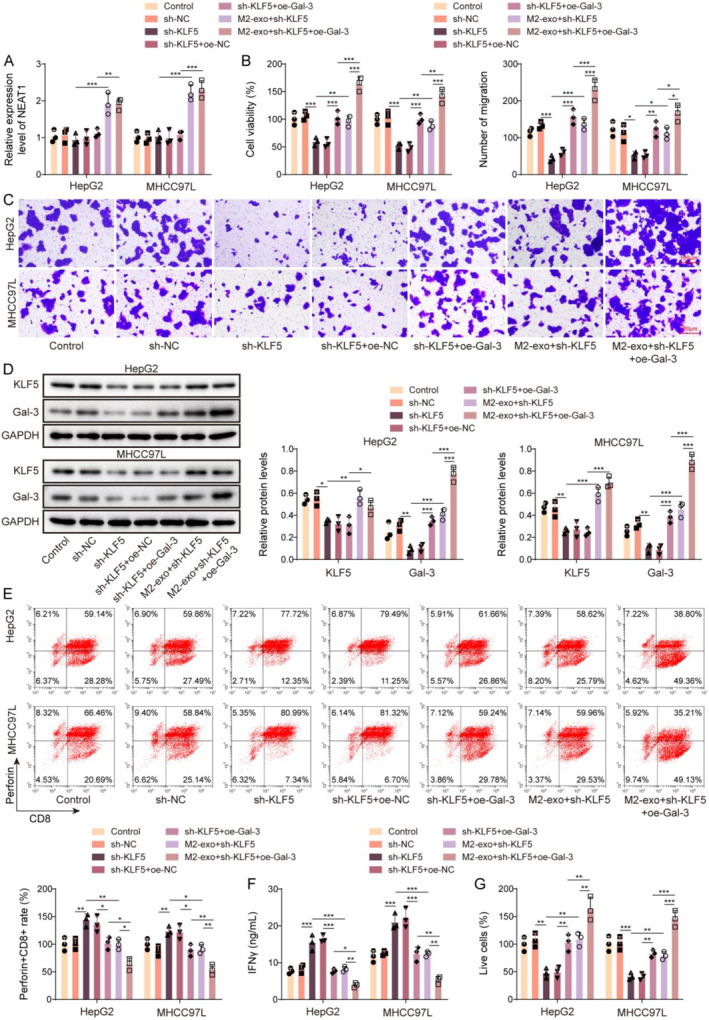
M2‐TAM‐derived exosomal NEAT1 upregulated galectin‐3 by recruiting KLF5 to promote HCC immune escape. HCC cells were treated with M2‐exos combined with sh‐KLF5 and oe‐Gal‐3 co‐transfection. (A) NEAT1 expression in HCC cells was examined using qRT‐PCR. (B) CCK8 assay was employed to detect HCC cell viability. (C) Cell migration was measured by Transwell assays (Scale bars = 100 μm). (D) Western blot was adopted to detect KLF5 and galectin‐3 protein levels in HCC cells. Isolate CD8^+^ T cells from PBMCs were co‐cultured with the above‐grouped HCC cells. (E) The activation of perforin^+^CD8^+^T cells was measured by flow cytometry. (F) IFNγ level in CD8^+^ T cells was examined using ELISA. (G) CCK8 assay was employed to detect HCC cell viability. The measurement data were presented as mean ± SD. All data was obtained from at least three replicate experiments. *p < 0.05, **p < 0.01, ***p < 0.001.

To investigate the interaction between NEAT1, KLF5, and galectin‐3 in M2‐exo‐mediated effect on HCC immune escape, HCC cells were treated with M2‐exos combined with sh‐KLF5 and oe‐Gal‐3 co‐transfection. As shown in Figure 6A, M2‐exos treatment significantly elevated NEAT1 expression in HCC cells, and sh‐KLF5 and oe‐Gal‐3 transfection had no significant effect on NEAT1 expression. In addition, KLF5 knockdown reduced the viability and proliferation of HCC cells. Galectin‐3 overexpression and M2‐exos alleviated the inhibition of KLF5 downregulation on the viability and proliferation of HCC cells. M2‐exos could further promote cell viability and proliferation in sh‐KLF5 and oe‐Gl3 co‐transfected HCC cells (Figure 6B–C). Moreover, the upregulation of galectin‐3 rescued the galectin‐3 level inhibited by sh‐KLF5 but had no effect on the KLF5 level. M2‐exos alleviated the inhibition of KLF5 knockdown on the expressions of KLF5 and galectin‐3, and galectin‐3 overexpression further upregulated galectin‐3 level in sh‐KLF5 and M2‐exos co‐treated HCC cells (Figure 6D). Isolated CD8^+^ T cells from PBMCs were co‐cultured with the above‐grouped cells. It turned out that KLF5 knockdown increased the activation of perforin^+^CD8^+^ T cells, galectin‐3 overexpression or M2‐exos treatment alleviated the promotion of KLF5 downregulation on the activation of perforin^+^CD8^+^ T cells, and further enhanced by M2‐exos (Figure 6E). Furthermore, KLF5 silencing increased IFNγ level in CD8^+^ T cells, whereas M2‐exos and galectin‐3 overexpression reversed the above effects. Galectin‐3 overexpression further downregulated the level of IFNγ in CD8^+^ T cells, which co‐cultured with KLF5 knockdown and M2‐exos co‐induced HCC cells (Figure 6F). It also turned out that KLF5 silencing enhanced the inhibitory effect of CD8^+^ T cells on HCC cell vitality, whereas M2‐exos and galectin‐3 overexpression reversed the above effect, and galectin‐3 overexpression further increased cell vitality in M2‐exos and sh‐KLF5 co‐treated HCC cells (Figure 6G). In conclusion, M2‐TAM‐derived exosomal NEAT1 increased galectin‐3 expression by recruiting KLF5 to promote HCC immune escape.

### 3.7 M2‐TAM‐derived exosomal NEAT1 promoted HCC tumor growth in vivo

**FIGURE 7:**
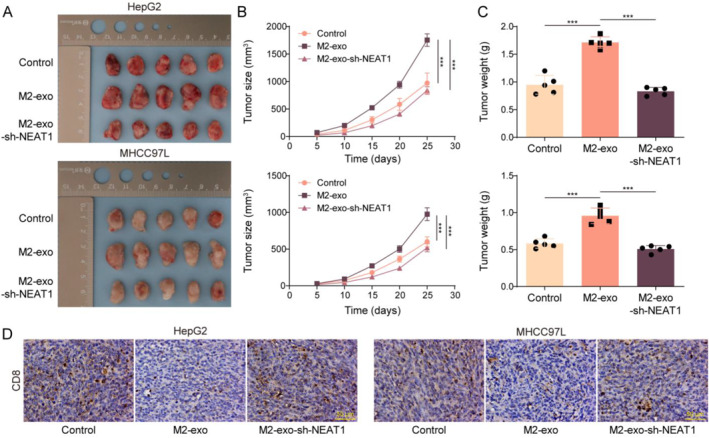
M2‐TAM‐derived exosomal NEAT1 promoted HCC tumor growth in vivo. C57BL/6 mice were injected with HCC cells (5 × 10^6^ cells per mouse) and treated with M2‐exo, M2‐exo‐shNEAT1, or saline every 3 days. (A‐C) The size and volume of tumors were presented. (D) CD8 level in tumor tissues was assessed by immunohistochemistry. The measurement data were presented as mean ± SD. *n* = 5. ***p < 0.001.

For xenograft studies, HCC tumor‐bearing mouse models were established. Starting on day 7, the tumor‐implanted mice were treated with M2‐exo, M2‐exo‐sh‐NEAT1, or saline every 3 days. As shown in Figures 7A–C, M2‐exo treatment markedly inhibited tumor growth, whereas this effect was abolished by NEAT1 downregulation. Additionally, the level of CD8 in tumor tissues was significantly reduced by M2‐exo treatment, whereas this change was eliminated by NEAT1 knockdown (Figure 7D). Collectively, M2‐TAM‐derived exosomal NEAT1 promoted HCC tumor growth in mice.

## 4 DISCUSSION

HCC is the most common type of primary liver cancer in the world.^26^ Immune escape, which refers to the acquired ability of HCC cells to avoid immune‐mediated lysis, is a significant element in HCC development.^27^ Repressing the immune escape of HCC cells might be a viable HCC treatment method. The current study found that M2‐TAM‐derived exosomal NEAT1 increased galectin‐3 expression by recruiting KLF5 to promote HCC immune escape.

The M1/M2 macrophage paradigm acts as a key part of tumor development. M1 macrophages have traditionally been thought to be anti‐tumor, but M2‐polarized macrophages contribute to a variety of pro‐tumorigenic outcomes in cancer, including immune suppression.^28^ In addition, M2‐TAMs infiltration usually suppresses the immune system and promotes cancer cell immune escape.^29^ Herein, it was observed that M2‐TAMs promoted HCC cell proliferation, migration and immune escape. Exosomes are small vesicles with phospholipid bilayers that serve as essential intercellular communication mediators.^30^ M2‐TAM‐secreted exosome transfer proteins, lncRNA, lipids and other molecules through the TME, endow cancer cells with different phenotypes, and contribute to immune escape.^29^ As evidence, M2‐TAM‐derived exosomal LINC01232 induced immune escape in glioma.^29^ It has been widely illustrated that NEAT1 plays a key role in promoting tumorigenesis.^14^, ^31^ NEAT1 is a key player in regulating immunity, including T cell activation.^32^ Notably, it was reported by Yi et al. that NEAT1 was a risk factor facilitating immune evasion in glioblastoma.^33^ In the current study, it was observed that NEAT1 was highly expressed in M2‐TAMs and M2‐exos. NEAT1 silencing reversed the promoting effect of M2‐exos on HCC cell viability, migration and immune escape. Collectively, M2‐TAMs promoted HCC immune escape by carrying NEAT1.

KLF5 is a critical oncogenic transcription factor in malignancies.^16^ Increasing evidence suggests that KLF5 can reshape the TME.^34^ A previous study described that KLF5 deletion could promote anti‐tumor immunity by enhancing the proliferation and function of CD4^+^ and CD8^+^ T cells.^19^ In the current research, it turned out that KLF5 was highly expressed in HCC cells. More importantly, NEAT1 directly interacted with KLF5. KLF5, being an efficient transcription factor, can recognize the GCCCGCCC pattern in gene promoters. Using the JASPAR database, we predicted KLF5 binding sites in the LGALS3 promotor region. It was subsequently confirmed that KLF5 could bind to the LGALS3 promotor and activate its transcriptional program. Galectin‐3 is proven to be a carbohydrate‐binding protein with regulatory involvement in tumor growth and metastatic processes.^35^ Notably, galectin‐3 overexpression was correlated with HCC metastasis and poor prognosis.^36^ The structural complexity of galectin‐3 allows it to bind with multiple molecules in the extracellular and intracellular milieu via protein‐protein and/or protein‐carbohydrate interactions and control several signaling pathways, some of which appear to be oriented at immune escape.^35^, ^37^ Along with PD‐1 and CTLA‐4, LAG3 is the most promising immune checkpoint. A high LAG3 level facilitates tumor development by suppressing the immune microenvironment.^38^ It has been extensively reported that galectin‐3 presents an immunosuppressive function in cancers by binding to LAG3.^23^ For example, galectin‐3 upregulation promoted immune escape in cancer by inhibiting CD8 T cells through binding to LAG3.^24^ Our results showed that M2‐TAM‐derived exosomal NEAT1 upregulated galectin‐3 in HCC cells by recruiting KLF5. As expected, KLF5 knockdown in HCC cells promoted T cell activation and prevented M2‐exos‐induced HCC immune escape, whereas galectin‐3 upregulation eliminated these effects mediated by KLF5 knockdown. All our results revealed that M2‐TAM‐derived exosomal NEAT1 upregulated galectin‐3 in HCC cells by recruiting KLF5 to promote T cell depletion and contribute to HCC immune escape.

In conclusion, our research study proved that M2‐TAM‐derived exosomal NEAT1 promoted immune escape in HCC by upregulating galectin‐3 by recruiting KLF5. These findings lay the theoretical groundwork for the development of innovative HCC treatments. But our research also has certain insufficiency. We didn't perform animal experiments to verify our experimental conclusion in vivo. We will conduct animal experiments in the future to make our research conclusions more credible.

## AUTHOR CONTRIBUTIONS

**Wei Yuan**: Conceptualization; formal analysis; investigation; methodology; validation; visualization; writing—original draft. **Qigang Sun**: Conceptualization; data curation; investigation; methodology; validation; visualization; writing—original draft. **Xiaodan Zhu**: Data curation. **Bo Li**: Formal analysis. **Yongping Zou**: Conceptualization; funding acquisition; project administration; supervision; writing—original draft; writing—review & editing. **Zhehao Liu**: Resources; software.

## CONFLICT OF INTEREST STATEMENT

All authors agree with the presented findings, have contributed to the work, and declare no conflicts of interest.

## ETHICS STATEMENT

The Ethics Committee at Hainan General Hospital, Hainan Affiliated Hospital of Hainan Medical University approved this study, and all subjects signed informed permission.

## Supporting information

Supporting Information S1

Figure S1

## Data Availability

All data generated or analyzed during this study are included in this published article.
