# Supplementary material for: M2‐polarized tumor‐associated macrophage‐secreted exosomal lncRNA NEAT1 upregulates galectin‐3 by recruiting KLF5 and promotes HCC immune escape
Source: J Cell Commun Signal. 2024 Dec 23;19(1):e12060. doi: 10.1002/ccs3.12060 (PMC11666343; doi:10.1002/ccs3.12060)
Supplement: Supplementary file 1 — Supporting Information S1 [file CCS3-19-e12060-s002.docx]

**Supplementary Figure 1 caption**

1. Western blot was employed to determine KLF5 and galectin-3 protein levels in HCC tumor tissues and adjacent normal tissues (n = 5). The measurement data were presented as mean ± SD. ***p < 0.001.
