# Supplementary figures and images for: M2‐polarized tumor‐associated macrophage‐secreted exosomal lncRNA NEAT1 upregulates galectin‐3 by recruiting KLF5 and promotes HCC immune escape
Source: J Cell Commun Signal. 2024 Dec 23;19(1):e12060. doi: 10.1002/ccs3.12060 (PMC11666343; doi:10.1002/ccs3.12060)

**Supplementary Figure 1**


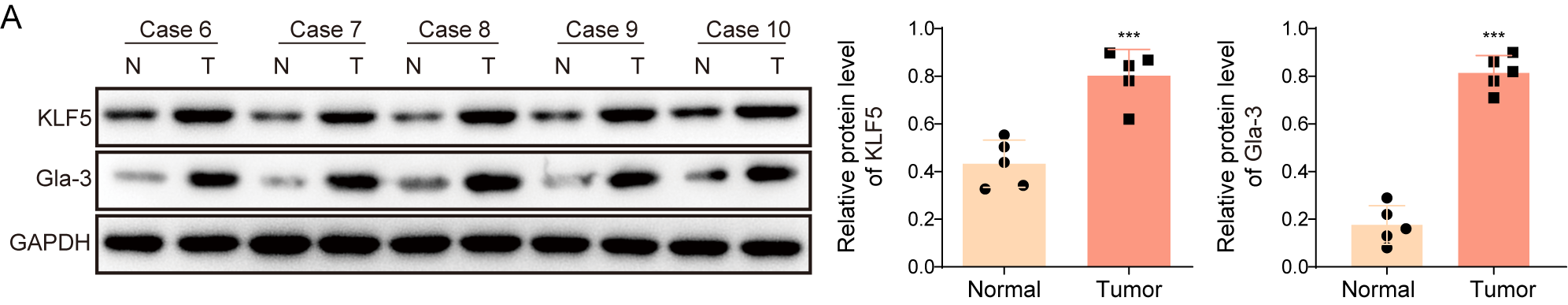

Supplement: Supplementary file 2 — Figure S1 [file CCS3-19-e12060-s001.docx]
